# Supplementary material for: Effect of Infraorbital and/or Infratrochlear Nerve Blocks on Postoperative Care in Patients with Septorhinoplasty: A Meta-Analysis
Source: Medicina (Kaunas). 2023 Sep 14;59(9):1659. doi: 10.3390/medicina59091659 (PMC10535682; doi:10.3390/medicina59091659)
Supplement: Supplementary file 1 [file medicina-59-01659-s001.zip › medicina-2601180-supplementary.pdf]

Table S1. Search terms and queries.

Database	Search	Search terms/queries
PubMed	#1	"Rhinoplasty"[Mesh]
	#2	"Rhinoplasty"[TW] OR "Rhinoplasties"[TW] OR "rhinoseptoplasty"[TW] OR "septorhinoplasty"[TW]
	#3 Combine	#1 OR #2
	#4	"Nerve Block"[Mesh]
	#5	"Nerve Block"[TW] OR "Block, Nerve"[TW] OR "Blocks, Nerve"[TW] OR "Nerve Blocks"[TW] OR "Nerve Blockade"[TW] OR "Blockade, Nerve"[TW] OR "Blockades, Nerve"[TW] OR "Nerve Blockades"[TW] OR "Chemical Neurolysis"[TW] OR "Chemical Neurolyses"[TW] OR "Neurolyses, Chemical"[TW] OR "Neurolysis, Chemical"[TW] OR "Chemodenervation"[TW] OR "Chemodenervations"[TW] OR "infraorbital nerve block"[TW] OR "infraorbital nerve"[TW] OR "infratrochlear nerve block"[TW] OR "infratrochlear nerve"[TW]
	#6	"Pain"[Mesh]
	#7	"Pain"[TW] OR "Pain, Burning"[TW] OR "Burning Pain"[TW] OR "Burning Pains"[TW] OR "Pains, Burning"[TW] OR "Suffering, Physical"[TW] OR "Physical Suffering"[TW] OR "Physical Sufferings"[TW] OR "Sufferings, Physical"[TW] OR "Pain, Migratory"[TW] OR "Migratory Pain"[TW] OR "Migratory Pains"[TW] OR "Pains, Migratory"[TW] OR "Pain, Radiating"[TW] OR "Pains, Radiating"[TW] OR "Radiating Pain"[TW] OR "Radiating Pains"[TW] OR "Pain, Splitting"[TW] OR "Pains, Splitting"[TW] OR "Splitting Pain"[TW] OR "Splitting Pains"[TW] OR "Ache"[TW] OR "Aches"[TW] OR "Pain, Crushing"[TW] OR "Crushing Pain"[TW] OR "Crushing Pains"[TW] OR "Pains, Crushing"[TW]
	#8 Combine	#4 OR #5 OR #6 OR #7
	#9 Combine	#3 AND #8
	#10 Combine	#9 NOT ("animals"[MeSH] NOT "Humans"[MeSH])

Database	Search	Search terms/queries
EMBASE	#1	"rhinoplasty"/exp
	#2	"Rhinoplasty":ti,ab,kw,de OR "Rhinoplasties":ti,ab,kw,de OR "rhinoseptoplasty":ti,ab,kw,de OR "septorhinoplasty":ti,ab,kw,de
	#3 Combine	#1 OR #2
	#4	"nerve block"/exp

	#5	"Nerve Block":ti,ab,kw,de OR "Block, Nerve":ti,ab,kw,de OR "Blocks, Nerve":ti,ab,kw,de OR "Nerve Blocks":ti,ab,kw,de OR "Nerve Blockade":ti,ab,kw,de OR "Blockade, Nerve":ti,ab,kw,de OR "Blockades, Nerve":ti,ab,kw,de OR "Nerve Blockades":ti,ab,kw,de OR "Chemical Neurolysis":ti,ab,kw,de OR "Chemical Neurolyses":ti,ab,kw,de OR "Neurolyses, Chemical":ti,ab,kw,de OR "Neurolysis, Chemical":ti,ab,kw,de OR "Chemodenervation":ti,ab,kw,de OR "Chemodenervations":ti,ab,kw,de OR "infraorbital nerve block":ti,ab,kw,de OR "infraorbital nerve":ti,ab,kw,de OR "infratrochlear nerve block":ti,ab,kw,de OR "infratrochlear nerve":ti,ab,kw,de
	#6	"pain"/exp
	#7	"Pain":ti,ab,kw,de OR "Pain, Burning":ti,ab,kw,de OR "Burning Pain":ti,ab,kw,de OR "Burning Pains":ti,ab,kw,de OR "Pains, Burning":ti,ab,kw,de OR "Suffering, Physical":ti,ab,kw,de OR "Physical Suffering":ti,ab,kw,de OR "Physical Sufferings":ti,ab,kw,de OR "Sufferings, Physical":ti,ab,kw,de OR "Pain, Migratory":ti,ab,kw,de OR "Migratory Pain":ti,ab,kw,de OR "Migratory Pains":ti,ab,kw,de OR "Pains, Migratory":ti,ab,kw,de OR "Pain, Radiating":ti,ab,kw,de OR "Pains, Radiating":ti,ab,kw,de OR "Radiating Pain":ti,ab,kw,de OR "Radiating Pains":ti,ab,kw,de OR "Pain, Splitting":ti,ab,kw,de OR "Pains, Splitting":ti,ab,kw,de OR "Splitting Pain":ti,ab,kw,de OR "Splitting Pains":ti,ab,kw,de OR "Ache":ti,ab,kw,de OR "Aches":ti,ab,kw,de OR "Pain, Crushing":ti,ab,kw,de OR "Crushing Pain":ti,ab,kw,de OR "Crushing Pains":ti,ab,kw,de OR "Pains, Crushing":ti,ab,kw,de
	#8 Combine	#4 OR #5 OR #6 OR #7
	#9 Combine	#3 AND #8
	#10 Combine	#9 NOT ('animal'/exp NOT 'human'/exp)

Database	Search	Search terms/queries
Cochrane Library	#1	[mh "Rhinoplasty"]
	#2	"Rhinoplasty":ti,ab,kw OR "Rhinoplasties":ti,ab,kw OR "rhinoseptoplasty":ti,ab,kw OR "septorhinoplasty":ti,ab,kw
	#3 Combine	#1 OR #2
	#4	[mh "Nerve Block"]
	#5	"Nerve Block":ti,ab,kw OR "Block, Nerve":ti,ab,kw OR "Blocks, Nerve":ti,ab,kw OR "Nerve Blocks":ti,ab,kw OR "Nerve Blockade":ti,ab,kw OR "Blockade, Nerve":ti,ab,kw OR "Blockades, Nerve":ti,ab,kw OR "Nerve Blockades":ti,ab,kw OR "Chemical Neurolysis":ti,ab,kw OR "Chemical Neurolyses":ti,ab,kw OR "Neurolyses, Chemical":ti,ab,kw OR "Neurolysis, Chemical":ti,ab,kw OR "Chemodenervation":ti,ab,kw OR "Chemodenervations":ti,ab,kw OR "infraorbital nerve block":ti,ab,kw OR "infraorbital nerve":ti,ab,kw OR "infratrochlear nerve block":ti,ab,kw OR "infratrochlear nerve":ti,ab,kw
	#6	[mh "Pain"]

	#7	"Pain":ti,ab,kw OR "Pain, Burning":ti,ab,kw OR "Burning Pain":ti,ab,kw OR "Burning Pains":ti,ab,kw OR "Pains, Burning":ti,ab,kw OR "Suffering, Physical":ti,ab,kw OR "Physical Suffering":ti,ab,kw OR "Physical Sufferings":ti,ab,kw OR "Sufferings, Physical":ti,ab,kw OR "Pain, Migratory":ti,ab,kw OR "Migratory Pain":ti,ab,kw OR "Migratory Pains":ti,ab,kw OR "Pains, Migratory":ti,ab,kw OR "Pain, Radiating":ti,ab,kw OR "Pains, Radiating":ti,ab,kw OR "Radiating Pain":ti,ab,kw OR "Radiating Pains":ti,ab,kw OR "Pain, Splitting":ti,ab,kw OR "Pains, Splitting":ti,ab,kw OR "Splitting Pain":ti,ab,kw OR "Splitting Pains":ti,ab,kw OR "Ache":ti,ab,kw OR "Aches":ti,ab,kw OR "Pain, Crushing":ti,ab,kw OR "Crushing Pain":ti,ab,kw OR "Crushing Pains":ti,ab,kw OR "Pains, Crushing":ti,ab,kw
	#8 Combine	#4 OR #5 OR #6 OR #7
	#9 Combine	#3 AND #8

Database	Search	Search terms/queries
Web of Science	#1	TS=("Rhinoplasty" OR "Rhinoplasties" OR "rhinoseptoplasty" OR "septorhinoplasty")
	#2	TS=("Nerve Block" OR "Nerve Blocks" OR "Nerve Blockade" OR "Nerve Blockades" OR "Chemical Neurolysis" OR "Chemical Neurolyses" OR "Chemodenervation" OR "Chemodenervations" OR "infraorbital nerve block" OR "infraorbital nerve" OR "infratrochlear nerve block" OR "infratrochlear nerve")
	#3	TS=("Pain" OR "Burning Pain" OR "Burning Pains" OR "Physical Suffering" OR "Physical Sufferings" OR "Migratory Pain" OR "Migratory Pains" OR "Radiating Pain" OR "Radiating Pains" OR "Splitting Pain" OR "Splitting Pains" OR "Ache" OR "Aches" OR "Crushing Pain" OR "Crushing Pains")
	#4	#2 OR #3
	#5 Combine	#1 AND #4

Database	Search	Search terms/queries
Google Scholar	#1	("Rhinoplasty" OR "Rhinoplasties" OR "rhinoseptoplasty" OR "septorhinoplasty")
	#2	("Nerve Block" OR "Nerve Blockade" OR "Chemical Neurolysis" OR "Chemodenervation" OR "infraorbital nerve" OR "infratrochlear nerve" OR "Pain" OR "Physical Suffering" OR "Ache")
	#3 Combine	("Rhinoplasty" OR "Rhinoplasties" OR "rhinoseptoplasty" OR "septorhinoplasty") AND ("Nerve Block" OR "Nerve Blockade" OR "Chemical Neurolysis" OR "Chemodenervation" OR "infraorbital nerve" OR "infratrochlear nerve" OR "Pain" OR "Physical Suffering" OR "Ache")
